# Within-Person Changes in Cancer Screening and Patient–Provider Communication Before and During COVID-19 in Kansas and Western Missouri

**DOI:** 10.21203/rs.3.rs-7689130/v1

**Published:** 2025-11-01

**Authors:** Carolyne Bukenya, Isuru Ratnayake, Lynn Chollet Hinton, Hope M. Krebill, Sam Pepper, Leah Lambart, Karla Goethem, Susan Beckman, Babalola Faseru, Ronald Chen, Dinesh Pal Mudaranthakam

**Affiliations:** University of Kansas Medical Center; University of Kansas Medical Center; University of Kansas Medical Center; University of Kansas Cancer Center; University of Kansas Medical Center; University of Kansas Cancer Center; University of Kansas Cancer Center; University of Kansas Cancer Center; University of Kansas Cancer Center; University of Kansas Cancer Center; University of Kansas Medical Center

**Keywords:** Cancer, Catchment Area, Cancer Survivor, COVID-19

## Abstract

**Background:**

The objective is to quantify within-person changes before versus during COVID-19 in (1) sources of health information, (2) patient–provider communication channels, and (3) time since last mammogram, Pap test, colonoscopy, and stool-kit screening among paired respondents in 123 counties within The University of Kansas Cancer Center’s (KUCC) catchment area.

**Aims:**

The study aims to assess changes in patients’ information seeking habits and evaluate whether screening intervals for mammograms, Pap tests, colonoscopies, and stool-kit use have lengthened or shortened.

**Methods:**

We conducted a paired pre–/during COVID-19 survey of the same patients across 123 counties in the KUCC catchment area. The survey instrument included items adapted from the Health Information National Trends Survey (HINTS) and the Behavioral Risk Factor Surveillance System (BRFSS) modules (validated/pilot-tested), along with investigator-developed items covering information sources, patient–provider communication, and timing of selected cancer screenings. Within-person changes were tested using McNemar’s tests for binary variables and Stuart–Maxwell tests for multi-category outcomes.

**Results:**

Among paired respondents (N = 751), information sources shifted from print to digital: internet use increased from 12.8% to 33.5% (+ 22.2%), email from 25.8% to 40.6% (+ 14.8%), while brochure use decreased from 43.1% to 26.6% (–16.5%; McNemar p < 0.050). Provider communication shifted toward EHR, email, text, and video (+ 25.7%, + 23.2%, + 24.9%, and + 10.0%, respectively; all p < 0.05). Screening timing changed significantly for mammography (χ^2^ = 27.0), colonoscopy (χ^2^ = 46.1), and stool test (χ^2^ = 25.1), but not for Pap test (χ^2^ = 3.07; p = 0.69).

**Conclusion:**

This study documents a shift from print to digital channels for health information and patient–provider communication, along with changes in screening timing for mammography, colonoscopy, and stool tests (with Pap timing unchanged). These findings highlight the importance of supporting multi-channel digital outreach to sustain preventive screening beyond the pandemic.

## Introduction

The United States spends more on healthcare than other high-income countries, driven largely by higher prices and administrative complexity [1, 2]. A 2023 BMJ report found that U.S. healthcare spending is more than double that of comparable countries, yet it yields worse outcomes across many health indicators [3]. The COVID-19 pandemic further precipitated sharp disruptions in preventive care, including documented declines in mammograms, colonoscopies, and Pap tests [4, 5]. These disruptions raise two system-level questions: (1) how did individual communication preferences with providers change during this period, and (2) did these shifts in communication correspond with changes in the timing of routine cancer screenings?

Due to limited access to routine services during COVID-19, it became crucial to understand changes in screening behaviors and communication preferences to guide effective re-engagement strategies. Prior work both predicted and confirmed substantial downturns in preventive oncology care during the pandemic, including missed visits and deferred diagnostic pathways [4–6]. Studies further suggested that even temporary reductions in screening and diagnostic testing could translate into excess mortality due to treatment delays [6]. At the same time, care delivery rapidly reconfigured toward remote modalities, with marked growth in portal use, email/text communication, and video encounters to sustain patient–clinician contact [7, 8]. What remains less well characterized especially in rural and frontier catchment areas, is the extent to which the same individuals altered how they sought health information (print vs. digital), how they communicated with providers (portal, email, text, video), and when they obtained key cancer screenings as the pandemic unfolded [9, 10].

We extended this literature with patient-level evidence from The University of Kansas Cancer Center (KUCC) catchment area by following the same individuals before and during COVID-19 across 123 counties in Kansas (105) and western Missouri (18). Of these, 91 Kansas counties and 14 Missouri counties are designated as rural or frontier by the U.S. Census and the Health Resources and Services Administration (HRSA), meaning that more than 85% of the KUCC catchment area is rural or frontier. This distinction is important, as these communities face limited healthcare access, greater travel distances, and lower digital readiness compared to urban centers. Our paired design analyzed behavioral changes beyond compositional shifts and linked two domains: (1) information-seeking behaviors (print vs. digital) and patient–provider communication channels (EHR portal, email, text, video), and (2) timing of mammograms, Pap tests, colonoscopies, and stool-kit screenings. Using within-subject tests, we quantified shifts in both communication and screening intervals to identify actionable levers for post-pandemic re-engagement and backlog recovery in settings with mixed digital readiness.

By examining within-person changes, this study builds on prior national and health system reports [7, 8] that documented broad shifts in patient communication behaviors during the COVID-19 pandemic. In contrast to those investigations, which relied on cross-sectional data, our paired survey design offers novel evidence on how individual patients adapted their communication preferences and cancer screening behaviors over time within the KUCC catchment area.

## Methods

### Survey instrument and validation

The survey instrument drew from established population-health items, including the Health Information National Trends Survey (HINTS) and the Behavioral Risk Factor Surveillance System (BRFSS), as well as guideline-aligned screening questions from the U.S. Preventive Services Task Force (USPSTF), with minor wording adaptations for local context. For example, diverse familial references (e.g., Papa, Grandpa, Grandma) were consolidated into unified categories such as Family to improve consistency and interpretability. The instrument was piloted to ensure content validity prior to fielding. Twenty questions were kept identical across both survey waves to enable within-person comparisons, consistent with approaches used in prior studies of behavioral change during the COVID-19 pandemic [9–10]. The Appendix includes the combined set of twenty survey questions used in this study.

### Study Design and Data Collection

We employed a survey-based approach to assess changes in patient communication preferences, cancer screenings, and preferred communication methods during the COVID-19 pandemic. Surveys were administered across the 123 counties in the KUCC catchment area, which is home to approximately 4.4 million residents, including rural and frontier communities with limited healthcare access.

Participants were recruited through an academic health system and primary care practices within two Accountable Care Organizations (ACOs), primarily serving rural areas. The first survey was mailed to a probability sample of 8,000 individuals, of whom 1,364 responded (17.9% response rate after excluding deceased or undeliverable addresses). The second survey (administered during COVID-19) included overlapping questions, enabling direct comparisons across time periods. Analyses were restricted to 751 respondents who completed both surveys, after excluding 189 (20%) with incomplete data. Twenty questions were identical across both surveys to facilitate within-person comparisons.

### Alignment of Responses

Some response options differed between surveys, so we created a crosswalk to align wording and categories. Race and ethnicity required standardization: in Survey One, detailed categories were collected and later collapsed by the consortium data committee (e.g., White, non-Hispanic; Black, non-Hispanic; Hispanic; American Indian; Other). In contrast, Survey Two presented fewer, broader categories, merging smaller groups such as *American Indian* into *Other*. To harmonize the datasets, Survey One responses were recoded to match Survey Two categories. In addition, open-ended fields were standardized using a controlled vocabulary (e.g., ‘Internet’ and ‘Social Media’ were collapsed into ‘Web Search’) to ensure comparability for paired analyses.

### Data Preparation and Statistical Analysis

Data were transformed from long to wide format to facilitate within-person comparisons. Missing or ‘NA’ responses were recoded as true missing values and handled consistently across analyses. For binary categorical outcomes, McNemar’s test was applied, while multi-category nominal outcomes were evaluated using the Stuart–Maxwell test for marginal homogeneity. Reporting and interpretation followed standard statistical texts, and results were considered statistically significant at a p-value threshold of 0.05. All analyses were conducted using SAS 9.3 and R 4.1 [9–11].

## Results

Participant demographics are summarized in Table 1. Across both survey waves, we analyzed data from 751 matched respondents (Survey 1: pre–COVID-19; Survey 2: during COVID-19), enabling within-person comparisons of communication preferences and screening behaviors over time. The cohort was predominantly female (66%) and White/non-Hispanic (92%), with a median age over 65 years. Nearly half of respondents held at least a bachelor’s degree, and most reported middle income.

**Figure 1:**
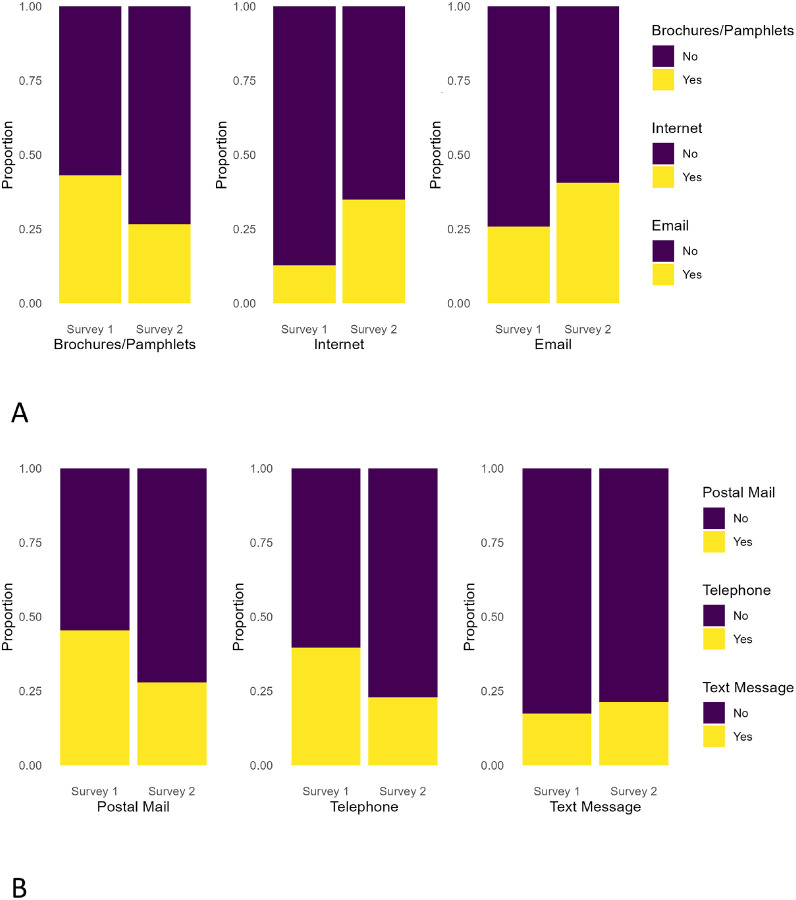
1A: Preferred methods (brochures/pamphlets, internet, and email) to receive health information fromproviders. 1B: Preferred methods (postal mail, telephone, and text message) to receive health information fromproviders.

The McNemar test revealed significant shifts in participants’ preferred methods for receiving health information from providers between Survey One (pre-pandemic) and Survey Two (during the pandemic). Significant changes were observed for brochures/pamphlets (χ^2^ = 56.03, p < 0.05), internet communication (χ^2^ = 101.68, p < 0.05), email communication (χ^2^= 33.98, p < 0.05), text messaging (χ^2^ = 4.48, p < 0.05), and telephone communication (χ^2^ = 52.12, p < 0.05), while traditional postal mail remained essentially unchanged. As shown in Fig. 1A, *preferred communication to receive health information from providers*, brochures and pamphlets showed little change, indicating continued reliance on traditional formats among some patients. By contrast, internet use and email communication increased substantially, reflecting a broader transition toward digital channels and highlighting notable changes in usage patterns. Similarly, Fig. 1B illustrates trends in participants’ preferred methods of receiving health information from providers. Postal mail showed minimal change across both surveys, while telephone calls declined noticeably. In contrast, text messaging showed a modest but clear increase, further suggesting a gradual shift toward digital communication methods.

**Figure 2:**
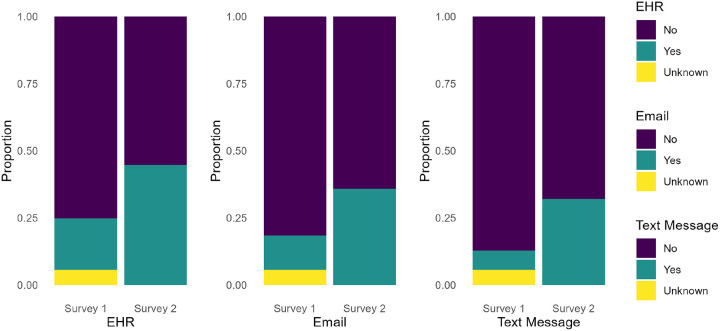
Preferred communication methods with health providers

Figure 2 highlights a shift in *preferred communication methods with healthcare providers*, focusing on electronic health record (EHR) portals, email, and text messaging to assess whether patients moved away from traditional approaches. Although some participants reported ‘*Unknown’* responses, suggesting uncertainty or lack of familiarity with these methods, the overall proportion of ‘*Yes’* responses for each digital channel increased, indicating growing comfort with these tools. Consistent with the McNemar test results, significant changes were observed for EHR (χ^2^ = 146.81, p < 0.05), email (χ^2^ = 108.11, p < 0.05), and text messaging (χ^2^ = 137.93, p < 0.05), each demonstrating notable increases between Survey One and Survey Two. Taken together, these findings underscore how patient–provider communication shifted further toward digital platforms during the pandemic.

**Figure 3:**
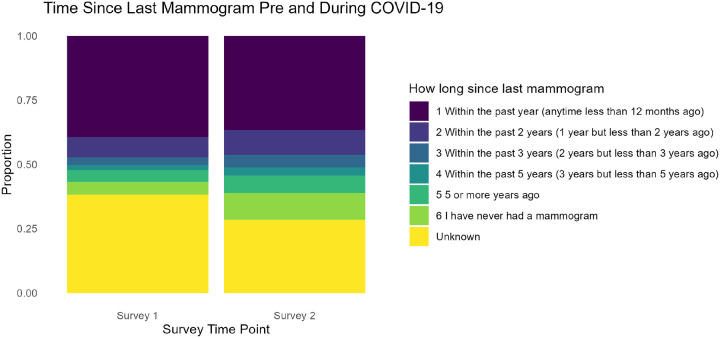
Changes in Mammogram Screening Timing Before and During COVID-19

Figure 3 illustrates the timing of mammogram screenings before and during the COVID-19 pandemic. Category 1— ‘*Within the Past Year’*—remained the most common response across both surveys, although it showed a slight decrease in Survey Two. In contrast, Category 6— ‘*I have never had a mammogram*’—increased slightly, though the small sample size in this group may reflect newly eligible individuals who had not yet been screened during the pandemic or other factors. Further analysis would be needed to determine whether this change was driven by pandemic-related barriers or by personal decisions. Overall, the Stuart–Maxwell results confirmed significant shifts in breast cancer screening patterns (χ^2^ = 27.03, p = 0.05), underscoring COVID-19’s impact on preventive care behaviors.

**Figure 4:**
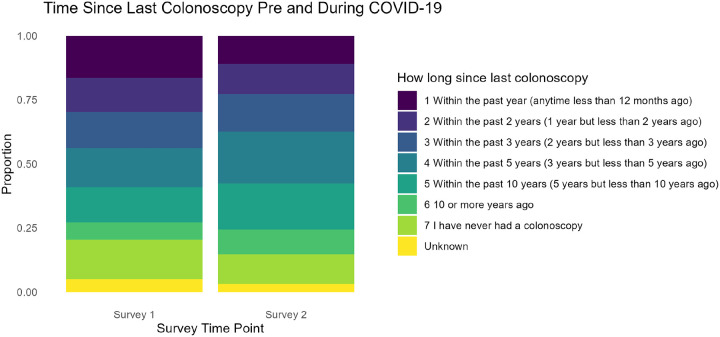
Changes in Colonoscopy Screening Timing Before and During COVID-19

Figure 4 displays the distribution of time since participants’ last colonoscopy, comparing Survey One (pre-pandemic) to Survey Two (during the pandemic). As with mammograms, we employed the Stuart–Maxwell test to evaluate whether these responses reflected statistically significant shifts over time. The results (χ^2^ = 46.14, p < 0.05) indicated that the distribution of colonoscopy timing changed notably. While some individuals maintained regular screening schedules, others reported postponements or delays. For example, categories such as ‘*Within the Past 5 Years*’ and ‘*10 or More Years Ago*’ showed marginal increases, suggesting that access challenges or hesitancy may have influenced participants’ colonoscopy decisions during the pandemic.

**Figure 5:**
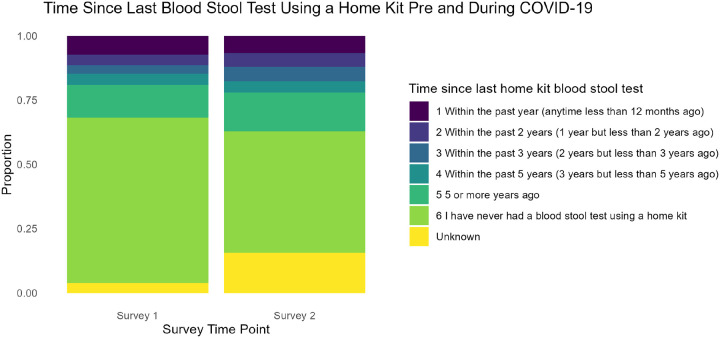
Changes in blood stool test using a home kit before and during COVID-19

Next, we examined changes in the timing of blood stool tests using home kits, as shown in Fig. 5. This comparison between Survey One (pre-pandemic) and Survey Two (during the pandemic) again employed the Stuart–Maxwell test to evaluate whether the distribution shifts were statistically significant. The results (χ^2^ = 25.14, p = 0.05) indicated notable changes over time. While the category ‘*Within the Past Year*’ showed a slight decrease, the proportion of participants reporting ‘*5 or More Years Ago*’ or ‘*I Have Never Had a Blood Stool Test Using a Home Kit*’ increased, suggesting potential delays or gaps in colorectal cancer screening during the pandemic.

**Figure 6:**
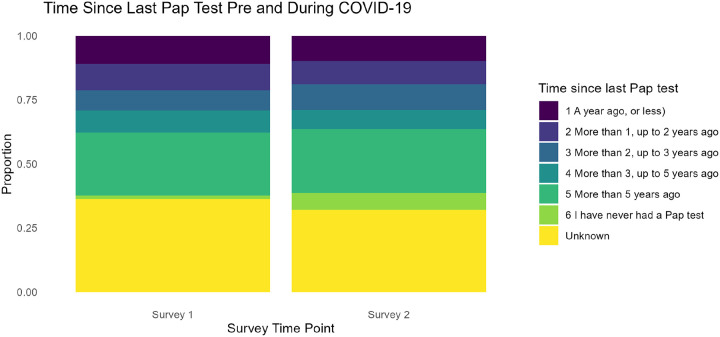
Changes in Cervical Cancer Screening Timing Before and During COVID-19

Figure 6 presents the distribution of time since participants’ last Pap test, comparing Survey One (pre-pandemic) to Survey Two (during the pandemic). In contrast to mammograms and colonoscopies, no substantial changes were observed in Pap test frequency between the two periods (χ^2^ = 3.07, p = 0.69). It is important to note that the average age of participants was 68 years (median = 70), and many women in this age group may no longer require or regularly receive Pap tests. This age distribution likely explains the absence of significant shifts, suggesting that the impact of the pandemic on cervical cancer screening may appear less pronounced in this older cohort compared with other screening modalities.

## Discussion

In this analysis, our goal was to evaluate changes during the COVID-19 pandemic in preferred methods for receiving health information, communication with healthcare providers, and cancer screening behaviors. Our findings provide meaningful insights for public health officials and healthcare organizations seeking to strengthen patient engagement and communication strategies, particularly in preparation for future health crises.

We observed notable shifts toward digital and telehealth communication channels during the pandemic, consistent with broader national trends. Although our survey did not directly capture participants’ motivations for delayed cancer screening, external evidence suggests that pandemic-related concerns and access barriers contributed to the widespread adoption of virtual care. This pattern aligns with rapid expansions in telehealth across the U.S., supported by McKinsey & Company projections of telehealth as a quarter-trillion-dollar post-pandemic industry and by expanded Medicare telehealth reimbursement policies. Patient and provider adoption has remained strong, with studies highlighting both the convenience and limitations of virtual care [11–13]. Large health systems, such as New York University (NYU) Langone, demonstrated the feasibility of scaling tele-visits from modest numbers per day to thousands [14]. Collectively, these developments underscore telehealth’s potential to sustain healthcare continuity even amid significant disruptions.

These insights from our study are valuable for understanding how public health communication preferences changed during this period. In an era of more robust technology, advanced communication methods such as email, EHR portals, and patient engagement platforms (e.g., Epic MyChart) have become increasingly pivotal. Epic MyChart, for example, enables patients and providers to remain engaged outside of office visits by providing continuous access to clinical information, scheduling tools, and post-visit follow-up. Our analysis demonstrates a shift from traditional mail toward more immediate digital communication [15], suggesting that pandemic conditions may have accelerated the adoption of responsive, technology-driven healthcare interactions.

Extending the conversation around digital communication and supporting online platforms will be crucial for enhancing patient–provider interactions and ensuring equitable access to care. According to the National Center for Health Statistics (NCHS), approximately 60% of adults in the United States seek medical information online [16]. Promoting telehealth visits can further leverage this broad internet use, offering patients quicker access to providers and more timely care.

Future longitudinal research will be essential to evaluate the lasting impact of these shifts. Such studies can clarify how digital methods and flexible care models may be effectively integrated into the healthcare delivery system to ensure sustained benefits and adaptability in the face of future challenges. In addition, research should assess how changing communication preferences affect healthcare delivery and identify strategies to support the additional provider time required, particularly in cases where this time is not currently reimbursable.

The overarching goal of these analyses was to identify trends in pre-pandemic versus during-pandemic changes, evaluate the impact of COVID-19 on cancer screenings, and examine preferred methods of communication with healthcare providers as well as preferred sources of health information. By employing these statistical tests, we sought to rigorously evaluate the data and draw meaningful conclusions about the pandemic’s impact on health behaviors and attitudes. These tests were selected because they are well-suited for comparing responses from the same participants at different time points, thereby capturing individual-level shifts rather than aggregate changes.

This broader perspective on patients’ preferences regarding health and medical information, cancer screening timing, and preferred methods of receiving information from providers offers a comprehensive understanding of how the pandemic influenced preventive care across multiple care settings. One key limitation of this study is its reliance on self-reported survey data, which may introduce reporting bias. In addition, the demographic composition of respondents was relatively homogenous, limiting the generalizability of findings to more diverse populations. Because the dataset was drawn primarily from a single geographic region, the results may not fully reflect nationwide trends. Finally, selection bias cannot be entirely ruled out, as participants who chose to respond may differ systematically from those who did not.

## Conclusion

Patient–provider communication will continue to evolve as technology becomes more readily available, with the goals of improving communication, increasing cancer screening, and reducing inefficiencies. Shifts in communication methods and screening practices will shape how care is delivered and received, underscoring the need for broader adoption of telehealth and other digital platforms. Implementing telehealth and electronic communication more widely has the potential to streamline healthcare interactions, enhance screening uptake, and reduce disparities in access. Future research should evaluate the impact of these technological advancements on patient outcomes and healthcare efficiency to ensure that such innovations meaningfully improve both patient care and system-wide effectiveness.

## Supplementary Files

This is a list of supplementary files associated with this preprint. Click to download.

- Appendix.docx

## Figures and Tables

**Table 1:** Summary of the Demographics

Characteristics	Sample Study (N = 751)	*Catchment Area (2019)
***Gender***			
Female	493 (65.6%)	2,279,197 (50.5%)
Male	250 (33.3%)	2,235,998 (49.5%)
Prefer not to answer	8 (1.1%)	NA
**Age**		
*0–18*	NA	1,210,987 (26.8%)
*19–64*	243(32.4%)	2,611,844 (57.9%)
*65+*	508 (67.6%)	692,364 (15.3%)
**Rurality (Urban/Rural Classification)**		
Rural	401(53.4%)	1,022,284 (22.6%)
Urban	350(46.6%)	3,492,911 (77.4%)
**Race**		
White, non-Hispanic	694(92.4%)	3,417,975(75.7%)
Black, non-Hispanic	15(1.9%)	362,967 (8.0%)
Hispanic	17(2.3%)	457,555 (10.1%)
Other	24(3.4%)	276,698 (6.1%)
**Education**		
Less than High School	14(1.9%)	408,237 (9.0%)
High School/GED	115(15.3%)	1,251,629 (27.7%)
Some College	214(28.5%)	1,411,274 (31.3%)
Bachelor’s/post-College	367(52.2%)	1,444,054 (32.0%)
Other	16(2.1%)	NA
**Household Income**		
$0 to $34,999	168(22.3)	1,297,576 (28.7%)
$35,000 to $49,999	75(10.0%)	622,545 (13.8%)
$50,000 to $74,999	138(18.4%)	847,238 (18.8%)
$75,000 to $99,999	120(16.0%)	606,717 (13.4%)
>$100,000	172 (22.2%)	1,141,119 (25.3%)
Other	83(11.1%)	NA
**Marital Status**				
Divorced/Separated	90(12.0%)	533,268 (11.8%)
Married/Partnered	492(65.5%)	2,354,372 (52.1%)
Other	8(1.1%)	N/A (0.0%)
Single	52(6.9%)	1,359,608 (30.1%)
Widowed	109(14.5%)	267,947 (5.9%)

* U.S. Census Bureau / American Community Survey (ACS) 2019 5-Year Estimates.

## Data Availability

Detailed data cannot be shared publicly to protect the privacy of individual participants. However, information to support the findings of these analyses is available by contacting the corresponding author. Upon reasonable request and understanding of the intended use of the data, the author will provide the requested information in a manner that continues to protect individual patient information.

## Abbreviations

EHR: Electronic Health Record
HINTS: Health Information National Trends
HRSA: Health Resources and Service Administration
MCA: Masonic Cancer Alliance
BRFSS: The Behavioral Risk Factor Surveillance System
KUCC: The University of Kansas Cancer Center
U.S. Census: United States Census
USPSTF: United States Preventive Services Task Force
